# Reconstruction of a Giant Congenital Melanocytic Nevus Defect With a Submental Flap in a Global Health Setting

**DOI:** 10.7759/cureus.16751

**Published:** 2021-07-30

**Authors:** Amanda L Brown, Ariel N Servin, Lawrence J McCarthy, Brian A Mailey

**Affiliations:** 1 Institute for Plastic Surgery, Southern Illinois University School of Medicine, Springfield, USA; 2 Plastic Surgery, N/A, San Diego, USA

**Keywords:** giant congenital melanocytic nevus, head and neck reconstruction, submental flap, perforator flap, congenital facial defect, global surgery, short term surgical missions, pediatric craniofacial surgery

## Abstract

Excision of a facial congenital melanocytic nevus (CMN) is a common reason for consultation in pediatric plastic surgery. Facial nevi are generally small and uncomplicated to remove and become more complex when large or giant. The available resources determine treatment and excision options. The indication for excision is generally based on esthetic criteria; however, the risk of melanoma increases with the nevi diameter. This patient with a giant CMN (GCMN) was encountered on an international medical mission trip. The palm-sized lesion spanned from her left zygomatic arch down to the jawline. Due to the esthetic impact and lack of resources to continue monitoring the lesion, complete excision was performed. The resultant defect was reconstructed with a pedicled submental flap. This article presents management and reconstruction of a facial GCMN encountered in the global setting and presents a brief literature review of GCMN.

## Introduction

Giant congenital melanocytic nevus (GCMN) of the face is a rare form of congenital nevus. The prevalence of newborns with CMN of any size and any bodily location ranges from 0.2% and 6% worldwide [1]. The incidence for a GCMN is estimated in less than 1:20,000 newborns. These lesions are typically hair-bearing and have major psychosocial impacts on the patient and family due to unpleasant appearance. Further, these lesions are associated with severe complications, including malignant melanoma and neurocutaneous melanosis. The quantitative risk of malignant transformation to melanoma is unknown. Some studies have reported that there could be a 1% chance for small and medium CMN and 5% for larger CMN, with about half the risk in the first few years of life [2]. Approximately 50% of melanoma develops by age two and 80% by age seven; thus, surgery at an early age is recommended [2-4]. Removal of a large or giant facial CMN requires a suitable donor site and often multiple procedures to achieve the best esthetic outcome. In third-world countries, close follow-up and repeat procedures are typically not available. Although several papers have described primary excision and closure of facial CMN, the authors are unaware of any reports that describe a pedicled submental flap to close the defect of a facial CMN in any setting. This technique provided a one-stage solution to close a defect that would otherwise necessitate tissue expanders or serial excisions performed in multiple stages. 

## Case presentation

**Figure 1:**
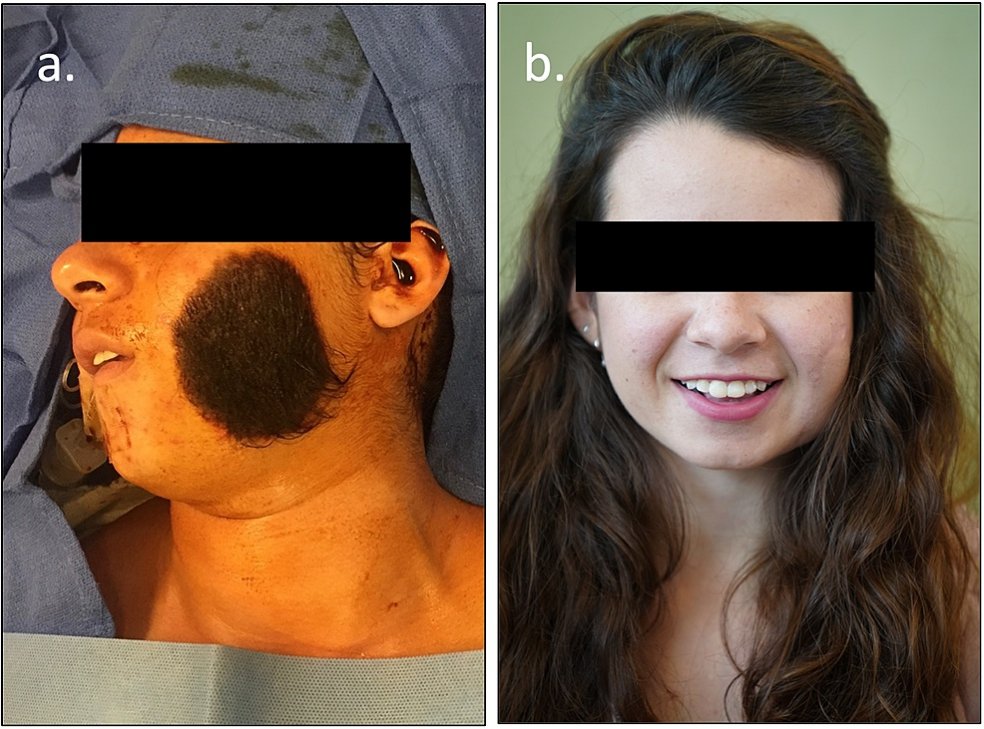
“Before and After” of a 15-year-old female with congenital hair-bearing melanocytic nevus overlying the left side of the face (a). Follow-up image after removal (b). This patient required one revision to reapproximate the edges due to distal dip necrosis with visible scarring on her right cheek.

A 15-year-old female presented with a 8 cm x 7 cm, ovoid-shaped, hairy GCMN on the left side of her face on a medical mission trip in Mexico. The palm-sized solitary lesion was present since birth and enlarged as she grew (Figure 1). An interview confirmed no personal or family medical history of melanoma or any form of cancer. The patient denied pain, pruritis, functional problems, or limitations in facial expression due to the lesion. Excision of the GCMN was performed due to the esthetic impact and lack of resources to continue monitoring the lesion.

**Figure 2:**
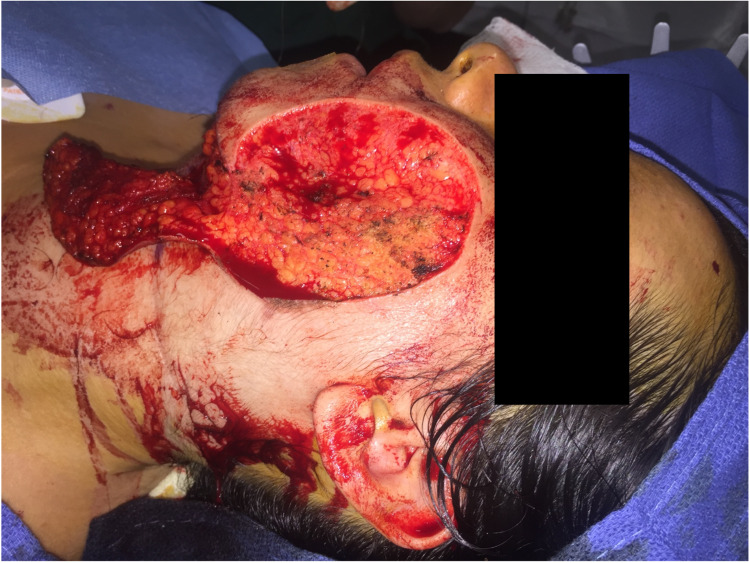
Intraoperative photograph after complete nevus excision. Note melanin deposition in the subcutaneous tissue at the level of the superficial muscular aponeurotic system. The patient did not have a diagnosis of malignancy and the melanin deposits were not removed to avoid potential damage to the facial nerve branches.

GCMN excisional surgery aims to remove the pigmented area as much as possible without sacrificing other functional structures in non-malignant cases. Nests of melanin deposits were embedded in the subcutaneous fat deep to the nevus (Figure 2). These were left in place to preserve the facial nerve branches, as the patient had no previous diagnosis of melanoma, and injuring her facial nerve would worsen outcome. Typically, the lesion would be biopsied to determine malignant patterns. In this global health setting, pathologic evaluation was not available; therefore, we provided the lesion in a specimen cup to the patient to have it evaluated separately. 

**Figure 3:**
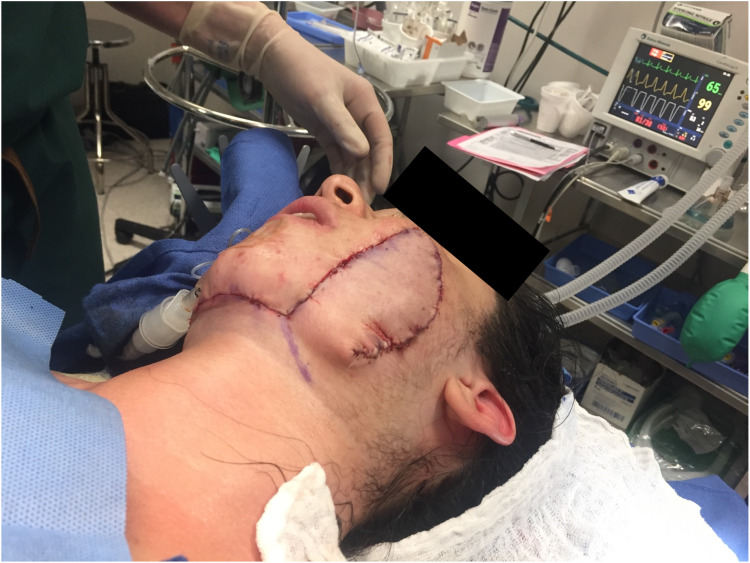
Immediate postoperative image after a left, pedicled submental flap was rotated onto the cheek to cover the defect.

Primary closure was not feasible due to the defect’s size and depth. We chose a pedicled submental flap to achieve the best esthetic outcome. The submental flap is indicated in defects of the oral cavity and the lower two-thirds of the face [5]. The flap was elevated distal to proximal in the submental plane across the midline until the contralateral anterior belly of the digastric muscle was encountered. The skin paddle was tailored to cover the defect adequately. An incision posterior to the mandibular angle was made, and a swatch of tissue was taken with the flap so the donor site would close primarily. The dissection was elevated beneath the platysma and carried to the defect. The submental cutaneous flap was rotated upward on the submental artery’s axis. The submental artery can be dissected backward to its origin at the facial artery to achieve flap advancement of another 1-2 cm to cover defects extending past the zygomatic arch; however, this was not necessary in this case (Figure 3). 

**Figure 4:**
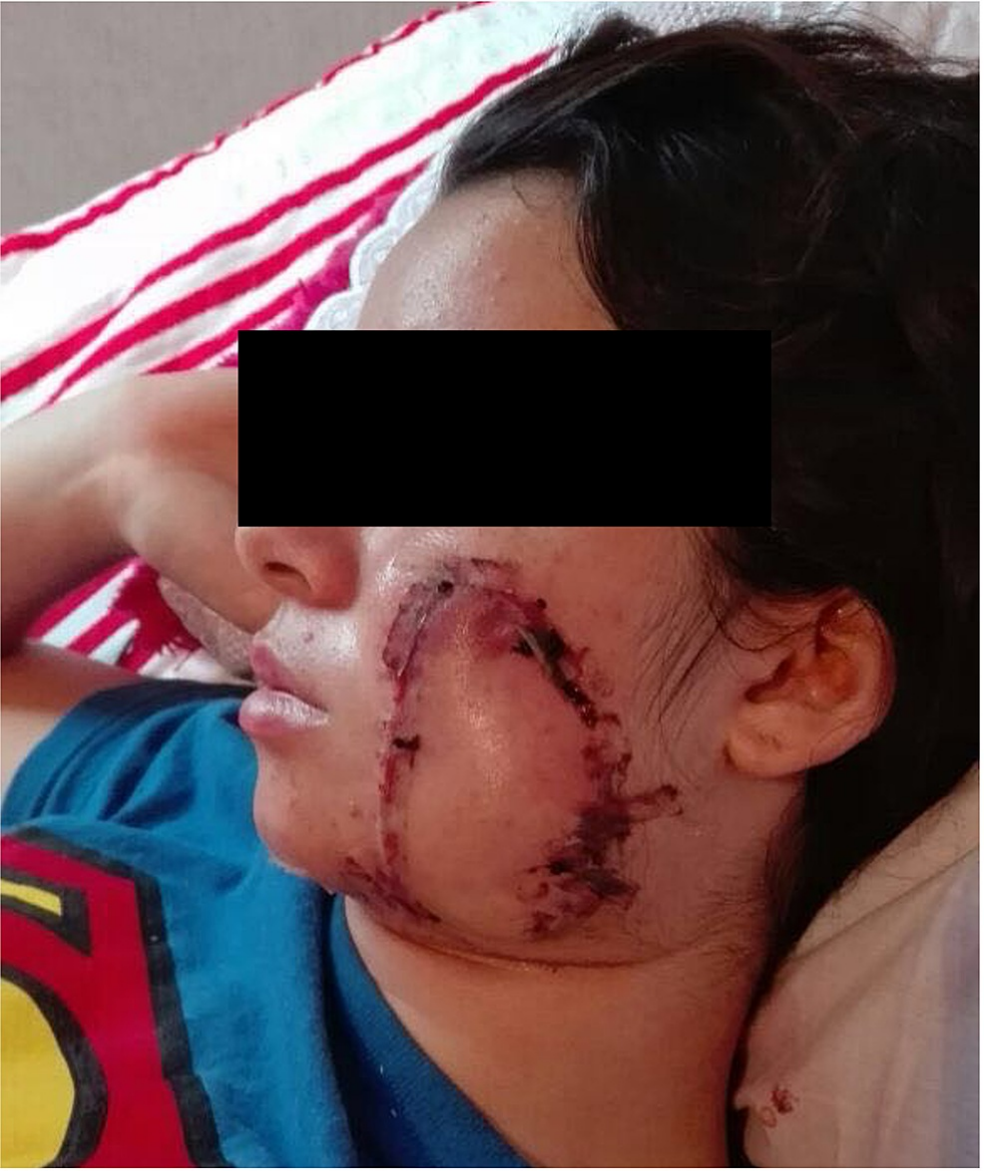
Early postoperative image demonstrating congestion of the distal aspect of the tip of the flap. The tip went on to necrose and heal in secondarily.

**Figure 5:**
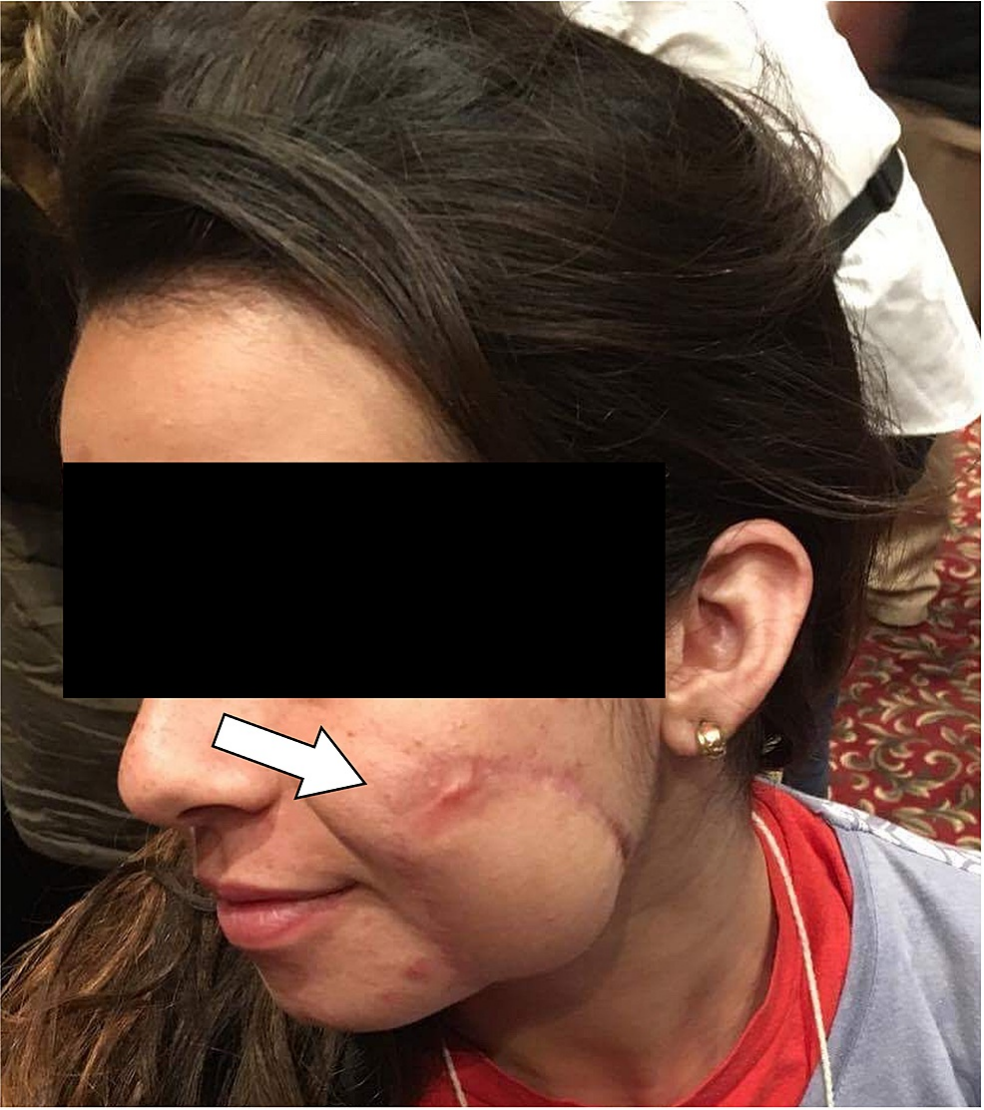
The same patient was encountered on a subsequent mission. The flap healed with widened scar at the distal tip.

**Figure 6:**
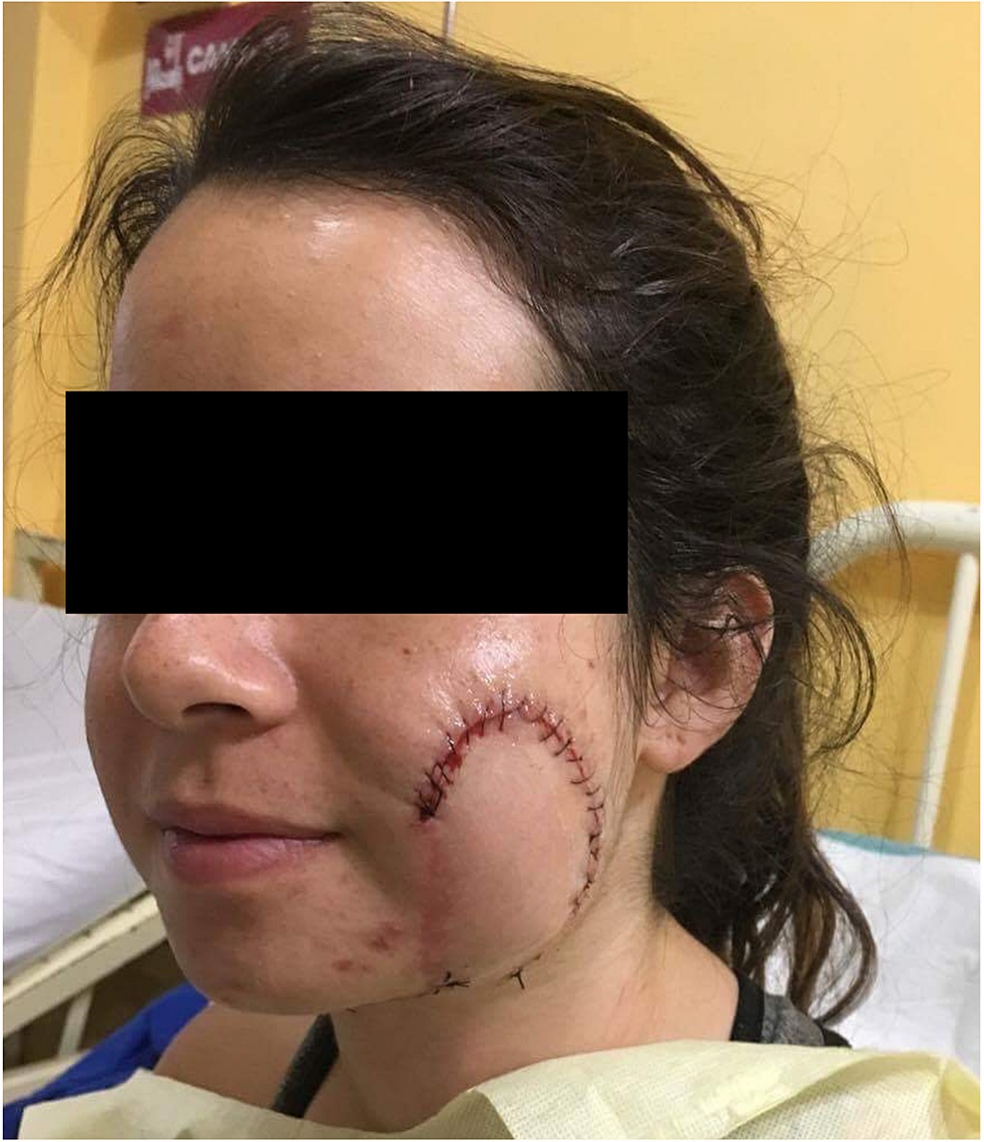
Subsequent revision and flap re-contouring approximated the flap edges to remove widened scar and more closely match the contour of the cheek.

Follow-up after short-term mission trips is typically available only for a short period, leading to uncertain long-term outcomes if the patient cannot follow up with a local provider. Luckily, this patient was re-encountered on a subsequent mission six months later, as partial tip necrosis of the flap occurred (Figure 4), leading to healing with a widened scar (Figure 5). The flap edge was recontoured (Figure 6).

## Discussion

The literature has variations in the size classification of CMN. Historically, Pers defined “giant” as palm-sized on the face and twice palm-sized elsewhere; some recommend the lesion to exceed >10 cm regardless of location; others classify a lesion that cannot be completely excised and closed primarily as “giant” [4,6].

Excision is the first-line treatment option for all large and giant hairy CMN due to the associated risk of malignant transformation, although the magnitude of this risk is debated. GCMN is typically limited to the skin but may invade the underlying structures, including bone and central nervous system [7]. In this case, the lesion did not invade the underlying structures deeper than the buccal subcutaneous adipose tissue, thus allowing proper excision. 

The magnitude of the defect necessitated flap closure to achieve the best esthetic result. The submental artery island flap is an efficient, reliable, esthetically matching option for soft tissue reconstruction of facial defects. Pedicled head and neck flaps have a robust blood supply and are very reliable tissue options that can be efficiently raised, even in third-world settings or with limited resources [8]. The laxity of the neck skin ("pinch test") determines the flap width allowing primary closure and can be as large as 18 cm x 7 cm. The submental artery flap is an efficient, reliable, esthetically matching option for soft tissue reconstruction of defects in the lower two-thirds of the face. In addition to distal tip necrosis, complications include facial nerve palsy, with an incidence of 0-17%, and damage to the marginal mandibular nerve [9]. A supraplatysmal approach can significantly lessen the risk of this damage [10]. The submental artery flap can be used in settings with limited resources, as it offers an efficient operation time, primary closure of donor site, minimal donor-site morbidity, and lower cost within a one-stage procedure.

## Conclusions

The case described in this report details the reconstruction of a giant congenital melanocytic nevus on the pediatric face. Many reports have outlined the primary excision and closure of facial CMN; however, the authors are unaware of any documentation describing a pedicled submental flap to close the defect of a facial CMN in any setting. With a complication of distal tip necrosis that was reapproximated on a subsequent mission, this patient had an overall acceptable outcome.
